# Highlighting Ophthalmic Adverse Effects Associated with Isotretinoin

**DOI:** 10.3390/life16091441

**Published:** 2026-08-29

**Authors:** Jason Timothy Pan, Benjamin S. Daniel, Benjamin Tan, Blanche X. H. Lim, Dedee F. Murrell, Stephanie L. Watson, Chris H. L. Lim

**Affiliations:** 1 Department of Ophthalmology, National University Health System, Singapore 119228, Singapore; jason.pan@u.nus.edu (J.T.P.); bentan78@gmail.com (B.T.); blanche_xh_lim@nuhs.edu.sg (B.X.H.L.); 2 Yong Loo Lin School of Medicine, National University of Singapore, Singapore 119077, Singapore; 3 Department of Dermatology, St. Vincent’s Hospital Melbourne, Victoria, VIC 3065, Australia; ben@drdaniel.com.au; 4 St. George Hospital, Kogarah, Sydney, NSW 2217, Australia; d.murrell@unsw.edu.au; 5 Save Sight Institute, Faculty of Medicine and Health, Sydney Medical School, The University of Sydney, Sydney, NSW 2000, Australia; stephanie.watson@sydney.edu.au; 6 Lions Eye Institute, Nedlands, WA 6009, Australia; 7 Singapore Eye Research Institute, Singapore 169856, Singapore

**Keywords:** dry eye disease, meibomian gland dysfunction, ocular surfaces disease, isotretinoin, photoreceptor, refractive surgery, corneal surgery

## Abstract

Isotretinoin is a highly effective treatment for moderate-to-severe acne vulgaris but is associated with a broad spectrum of ophthalmic adverse effects. While dry eye disease secondary to meibomian gland dysfunction is the most common ocular complication, less familiar manifestations include nyctalopia, corneal abnormalities, retinal changes, idiopathic intracranial hypertension, and optic neuritis. Current evidence suggests that isotretinoin induces structural and functional changes in the meibomian glands, resulting in tear film instability and evaporative dry eye disease. Although many of these changes appear reversible following treatment cessation, some patients may experience persistent symptoms. This review was conducted as a structured narrative review of the ophthalmic adverse effects associated with systemic isotretinoin use. A literature search was performed using PubMed and Embase for articles published from inception to 1 June 2026. Nyctalopia is a well-established adverse effect attributable to rod photoreceptor dysfunction and may have important implications for occupations requiring optimal night vision. Corneal remodelling and ocular surface dysfunction are particularly relevant in contact lens wearers and patients considering refractive and corneal surgery, for instance corneal transplants. Rare but potentially sight-threatening neuro-ophthalmic complications have also been reported. Clinicians prescribing isotretinoin should routinely enquire about visual symptoms and maintain a low threshold for ophthalmic referral. Early recognition and collaborative management between dermatologists, general practitioners, and eye care practitioners are essential to minimise visual morbidity, promote ocular comfort and optimise patient outcomes. This review aimed to provide an updated, clinically focused synthesis of the ophthalmic adverse effects of isotretinoin, emphasising pathophysiological mechanisms, clinical presentation, evidence supporting reversibility, and practical recommendations for ophthalmologists, dermatologists, and primary care clinicians.

## 1. Introduction

Isotretinoin, also known as 13-cis-retinoic acid, is a commonly utilised drug by dermatologists and general practitioners (GPs) in the treatment of acne vulgaris. Isotretinoin induces the expression of tumour necrosis factor-related apoptosis-inducing ligand (TRAIL), insulin-like growth factor binding protein-3 (IGFBP3) and neutrophil gelatinase-associated lipocalin (NGAL) to exert apoptotic effects on sebocytes [1,2]. While isotretinoin is mainly indicated in moderate-to-severe acne vulgaris [3,4,5], it is also used to manage folliculitis, some types of alopecia, cutaneous lupus, hidradenitis suppurativa, and rosacea [6,7]. Although generally well-tolerated, isotretinoin is associated with a broad spectrum of adverse effects involving the mucocutaneous, musculoskeletal, neurological, psychiatric, and ocular systems. These complications may adversely affect visual function, ocular comfort, and quality of life, and, in selected occupations such as aviation, have important occupational implications. Despite increasing recognition of isotretinoin-associated ocular toxicity, much of the available literature comprises observational studies and case reports, with inconsistent findings regarding the reversibility, pathophysiological mechanisms, and long-term outcomes of several ophthalmic complications. Existing reviews have largely focused on individual ocular manifestations or specific specialties, with limited emphasis on integrating current evidence into practical recommendations for clinicians involved in prescribing isotretinoin. The aim of this structured narrative review is to synthesise the current evidence regarding the ophthalmic adverse effects of systemic isotretinoin, including the underlying mechanisms, clinical manifestations, diagnostic approaches, reversibility, and management strategies. Emphasis is placed on clinically relevant recommendations for ophthalmologists, dermatologists, general practitioners, and other healthcare professionals involved in the care of patients receiving isotretinoin.

## 2. Methods

This review was conducted as a structured narrative review of the ophthalmic adverse effects associated with systemic isotretinoin use. A literature search was performed using PubMed and Embase for articles published from inception to 2026. The search string was as follows: (“Isotretinoin”[Mesh] OR isotretinoin[tiab] OR “13-cis-retinoic acid”[tiab] OR “13 cis retinoic acid”[tiab]) AND (“Eye”[Mesh] OR ocular[tiab] OR ophthalmic[tiab] OR eye[tiab] OR “ocular surface”[tiab] OR “dry eye”[tiab] OR “dry eye disease”[tiab] OR “meibomian gland dysfunction”[tiab] OR MGD[tiab] OR meibography[tiab] OR “meibomian gland”[tiab] OR cornea*[tiab] OR retina*[tiab] OR “retinal nerve fibre layer”[tiab] OR “retinal nerve fiber layer”[tiab] OR RNFL[tiab] OR GCIPL[tiab] OR nyctalopia[tiab] OR “night blindness”[tiab] OR “optic neuritis”[tiab] OR “intracranial hypertension”[tiab] OR papilledema[tiab] OR papilloedema[tiab] OR “visual function”[tiab]), and (‘isotretinoin’/exp OR isotretinoin:ti,ab OR ‘13-cis-retinoic acid’:ti,ab OR ‘13 cis retinoic acid’:ti,ab) AND (‘eye’/exp OR ‘eye disease’/exp OR ‘ocular surface’/exp OR ‘dry eye’/exp OR ‘meibomian gland dysfunction’/exp OR ‘meibography’/exp OR ‘cornea’/exp OR ‘retina’/exp OR ‘retinal nerve fiber layer’/exp OR ‘night blindness’/exp OR ‘optic neuritis’/exp OR ‘intracranial hypertension’/exp OR ocular:ti,ab OR ophthalmic:ti,ab OR eye:ti,ab OR ‘dry eye’:ti,ab OR ‘dry eye disease’:ti,ab OR ‘meibomian gland dysfunction’:ti,ab OR MGD:ti,ab OR meibography:ti,ab OR cornea*:ti,ab OR retina*:ti,ab OR RNFL:ti,ab OR GCIPL:ti,ab OR nyctalopia:ti,ab OR ‘night blindness’:ti,ab OR ‘optic neuritis’:ti,ab OR ‘intracranial hypertension’:ti,ab OR papilledema:ti,ab OR papilloedema:ti,ab). Reference lists of relevant articles were also manually screened to identify additional publications. Hand-searching of references from key publications was done to identify additional studies. Original research articles, observational studies, clinical trials, case series, case reports, review articles, and relevant clinical guidelines reporting ophthalmic adverse effects associated with systemic isotretinoin were considered for inclusion. Articles not published in English, studies involving topical retinoids alone, and reports without ophthalmic outcomes were excluded. This search strategy was developed in accordance with the framework from the Scale for the Assessment of Narrative Review Articles (SANRA). Titles and abstracts identified through the database searches were screened for relevance by Pan JT. Potentially relevant articles were subsequently assessed in full text against the predefined inclusion criteria. Studies were included if they reported clinical, structural, functional, mechanistic, or management-related evidence concerning ophthalmic effects of isotretinoin. Articles were excluded if they were unrelated to ophthalmic outcomes, did not provide relevant clinical or mechanistic information, or were non-English publications. Given the narrative nature of the review, study selection was performed by a single reviewer, with uncertainty regarding eligibility resolved through discussion among the authors, and no formal quality assessment or risk-of-bias analysis was performed.

## 3. General Side Effect Profile

Isotretinoin’s side effects are broad but generally manageable, enabling patients to continue their use (Table 1). The most common side effects are mucocutaneous, of which cheilitis and xerosis are the most frequent [8]. Other dermatologic side effects include transient worsening of acne vulgaris, photosensitivity, pruritus and desquamation, representing 65% of all reported side effects [3]. While these effects are generally predictable and dose-dependent, isotretinoin can uncommonly induce severe acne [9,10]. Isotretinoin is also teratogenic with congenital malformations reported and should be avoided at all stages of pregnancy [11,12,13]. Hyperlipidaemia [14,15,16] and transaminitis [17] are uncommon laboratory changes with isotretinoin, which can be monitored with baseline and subsequent investigations [18]. Retinoid hyperostosis (i.e., excessive bone formation) and premature epiphyseal closure, which may impair longitudinal bone growth in skeletally immature patients, have been uncommonly reported [19,20,21]. Associations between isotretinoin use and gastrointestinal [22,23] or psychiatric effects [24,25,26] remain inconclusive due to conflicting evidence.

## 4. Ophthalmic Side Effects

While general practitioners, dermatologists and cosmetic physicians may be aware of common systemic side effects, isotretinoin is also associated with a range of ophthalmic manifestations and side-effects. Ocular side effects can manifest either early or late during treatment and demonstrate dose-dependent characteristics. These ocular adverse effects range from common symptoms that impair ocular comfort and quality of life, such as dry eye disease, to rare but potentially sight-threatening complications including idiopathic intracranial hypertension, optic neuritis, and retinal vascular events (Table 2). These effects are critical to identify as they can impact visual function as well as ocular comfort. Practitioners should proactively enquire about related symptoms, and consider an early referral to an eye care practitioner with the onset of symptomology, to exclude ocular side effects and complications when on treatment with isotretinoin.

### 4.1. Nyctalopia (Night Blindness)

Nyctalopia describes difficulty in visualising under dim lighting conditions due to an inability to adapt from light to darkness, with photopic vision largely unaffected [31]. Nyctalopia arises from vitamin A deficiency which results in impaired rod-mediated dark adaptation and night vision [32]. Studies suggest that isotretinoin-associated nyctalopia arises from rod photoreceptor dysfunction, supported by electrophysiological evidence [33,34,35], and is therefore regarded a ‘certain’ side effect [28]. Purported mechanisms include interference with endogenous vitamin A processing in the retina [34], inhibition of 11-cis-retinol dehydrogenase in the retinal pigment epithelium [36], and toxic effects on the rod cells of the retina [37]. Some have proposed RNFL thinning as a separate mechanism for nyctalopia [38], while other studies have reported no significant relationship [39]. Nyctalopia associated with isotretinoin use seems to display a dose-dependent effect with higher doses conferring higher risk [33,37]. The reported dose–response relationship is supported by the pharmacological action of isotretinoin on vitamin A metabolism within the retina. By disrupting chromophore recycling and slowing rhodopsin regeneration, higher isotretinoin exposure may result in greater impairment of rod-mediated dark adaptation, providing a mechanistic basis for dose-dependent nyctalopia [40]. Symptoms typically appear within 2 weeks of treatment initiation in reported cases [34]. Its effects are generally reversible upon cessation of therapy, but permanent loss of dark adaptation can occur [28,33]. Vigilant monitoring and early detection in patients taking isotretinoin is thus important to minimise visual loss. The potential effects of isotretinoin on visual function and neuropsychiatric performance are of particular concern in aviation medicine, where optimal visual and cognitive function are essential for flight safety. In particular, isotretinoin’s impact on night vision as well as its possible psychiatric effects have led to civil and military aviation authorities worldwide either prohibiting its use or imposing operational restrictions on pilots who are on isotretinoin treatment. Of note, the UK Civil Aviation Authority (CAA) prohibits its use in pilots and stipulates a 2-week treatment cessation before pilots can receive medical certification. The US Federal Aviation Administration (FAA) mandates a 2-week waiting period after starting isotretinoin treatment, following which pilots may receive medical certification but remain restricted to daytime flying [36]. Military aviation authorities worldwide strictly prohibit isotretinoin use in military pilots as subtle impairments in visual performance can jeopardise operational safety in military aviation operations. It is imperative that medical practitioners are aware of the implications that commencement of isotretinoin treatment may have on certain vocations.

### 4.2. Retinal Adverse Effects

Current evidence regarding isotretinoin’s effects on the RNFL and ganglion cell-inner plexiform layer (GCIPL) remains inconsistent (Table 3). While Yilmaz et al. observed significant RNFL (particularly in the temporal quadrant) and GCIPL thinning as early as 3 months post initiation of systemic isotretinoin [38], others have observed no significant changes in RNFL or macula thickness over similar time frames [41,42], or even after 12 months of systemic isotretinoin at a cumulative dose of 120–150 mg/kg [43]. Ucak et al. reported selective thinning in lower temporal quadrants of the RNFL; this finding has not been replicated and an underlying pathophysiological explanation has not been established [44]. These studies are limited by their smaller sample sizes and inconsistent findings. As such, routine retinal structure monitoring in isotretinoin users is not supported by evidence. From an electrophysiological standpoint, rod cone photoreceptor dysfunction has been reported, and the abnormal electroretinogram findings accounted for by isotretinoin’s competitive inhibition of normal retinol binding sites [45]. Other rarely reported retinal pathologies may result in permanent vision loss, include retinal detachment [46], central retinal vein occlusion [47], and pre-macular haemorrhage [48].

### 4.3. Dry Eye Disease

**Figure 1:**
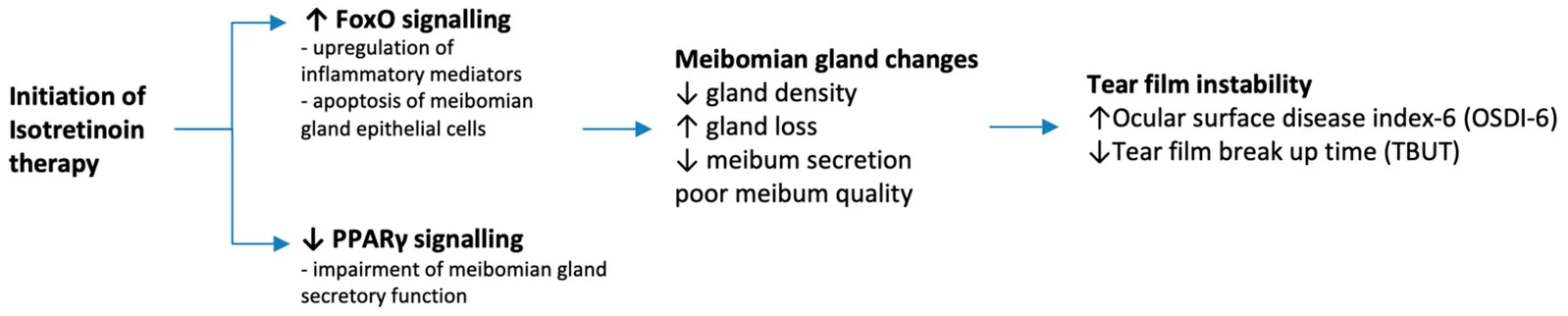
Suggested biochemical mechanisms of dry eye disease in isotretinoin users.

The normal tear film consists of a mucoaqueous layer, along with a lipid layer, with the meibomian glands responsible for the production of the meibum which constitutes this lipid layer [49]. DED can be due to tear film deficiencies—further classified as evaporative or aqueous-deficient, eyelid abnormalities (including abnormal blink rate, lid margin abnormalities and lid closure) and ocular surface abnormalities. Patients suffering with DED experience negative impacts on their quality of life [50]. The most common ocular side effect is dry eye disease (DED) due to meibomian gland dysfunction (MGD) and blepharoconjunctivitis [27,28]. In the largest compilation of reports on drug-associated ocular side effects, DED and blepharoconjunctivitis were considered ‘certain’ ocular surface alterations attributed to the use of isotretinoin [29]. In one of the earliest reports on isotretinoin associated DED, a third of patients reported DED symptoms after a 16-week treatment course [30]. Several mechanisms of DED have been proposed in patients on isotretinoin (Figure 1). Isotretinoin has been reported to induce FoxO signalling, resulting in upregulation of inflammatory mediators and apoptosis of meibomian gland epithelial cells [1]. The consequent reduction and dysfunction in meibum secretion destabilises the tear film lipid layer, leading to evaporative DED [51,52]. More recently, isotretinoin-induced disruption of the PPARγ signalling pathway have been implicated in the impairment of meibomian gland secretory function, contributing to MGD, tear film instability, and evaporative DED [53]. While other studies have suggested blepharoconjunctivitis [54] and reduced corneal sensitivity [55] as factors accounting for DED symptoms, patients on isotretinoin who report dry eye symptoms invariably suffer from some degree of MGD [56].

#### 4.3.1. Effects on Meibomian Glands

The meibomian glands are modified sebaceous glands that lie within the eyelid tarsal plate and secrete onto the eyelid margin. Their functional similarity to cutaneous counterparts and embryological derivation from ectoderm likely underlies their susceptibility to isotretinoin-induced dysfunction. They are best assessed by eye care practitioners using meibography with measures of structural (i.e., meibomian gland loss (MGL) and lid margin abnormality score (LAS)) and functional (i.e., meibum expressibility score (MES), meibum quality score (MQS)) changes [57] (Table 4). Early reports demonstrated decreased density and atrophy of the meibomian glands with isotretinoin therapy [58], as well as the decline in functional meibomian gland parameters [56,59,60]. These reports did not include sufficient post-therapy follow-ups to determine reversibility upon therapy cessation. In a prospective longitudinal study, Zakrzewska et al. demonstrated significant increases in MGL, deterioration in gland function (MQS and MES), and worsening ocular symptoms during isotretinoin therapy, with partial recovery of both structural and functional parameters one month after treatment cessation, although gland loss remained above baseline, which suggests some reversibility post-treatment [61]. Deterioration of secretory function (MES, MQS) has recently been found to correlate with gland loss during treatment, and both show improvement after treatment cessation [61,62]. Similarly, LAS not only increases during treatment and correlates well with MGL and MQS, but also significantly lowers after cessation of therapy, implying LAS reversibility [59,61]. While most studies attribute meibomian gland changes solely to isotretinoin initiation, few account for the effect of pre-existing dermatological conditions on meibomian glands. In treatment naïve populations, lid scores and meibomian glands scores have been found to be higher in patients with pre-existing acne vulgaris [62]. Similarly, treatment-naïve rosacea patients also demonstrate significantly greater lid margin abnormalities and MGL than controls [63]. A case can therefore be made for early ophthalmic evaluation for identification of MGD before the initiation of isotretinoin. Rarely, LAS may persist as long as 12 months after cessation of isotretinoin [64]. Moy et al. described three cases of isotretinoin-associated ocular surface disease with significant dry eye symptoms despite preserved meibomian gland morphology on meibography [52]. However, the timing of meibography relative to treatment initiation was not reported, and functional impairment may precede detectable structural changes over shorter follow-up periods. Overall, long-term outcome evidence is limited, and further prospective cohort studies are recommended.

#### 4.3.2. Effects on the Tear Film

The tear film is assessed using fluorescein staining and tear film break-up time (TFBUT), which reflects stability of the tear film [65]. Aqueous tear production can be quantified through assessments such as the Schirmer’s test or Phenol red thread test, along with clinical quantification of the tear film reservoir [66]. Dry eye symptom burden can be measured using validated patient-reported outcome measures [67], with the TFOS DEWS III guidelines recommending the streamlined ocular surface disease index-6 (OSDI-6) questionnaire (with a total sum ≥4 being suggestive of DED) as a standardised screening tool for symptom assessment [68]. Other indicators of ocular surface health include blink rate and usage of artificial tears. While patients with acne vulgaris, particularly nodulocystic acne, exhibit poorer baseline ocular surface parameters (e.g., TBUT and OSDI) and increased dry eye symptoms compared with healthy controls [69], and are predisposed to a poorer ocular surface, most studies demonstrate a significant increase in OSDI scores during treatment with isotretinoin [56,59,62].

The association between the severity of dry eye symptoms and severity of meibomian gland morphological change is equivocal. Caglar et al. reported increased OSDI scores and reduced TFBUT during treatment, as well as a significant correlation between the grade of MGD and TFBUT [56]. These associations suggest MGD as a significant contributing factor behind DED in isotretinoin and imply that the OSDI is a sufficient tool in monitoring MGD progression. However, Duzgun et al. reported worsening OSDI scores and meibum expression during treatment. While meibography scores correlated positively with LAS and MES, no correlation with other ocular surface parameters including TFBUT and Schirmer’s test was found [59]. Few studies have performed post-cessation evaluation of signs and symptoms, and thus the reversibility of ocular surface parameters after stopping isotretinoin is uncertain. Zakrzewska et al. observed a significant increase in OSDI scores after 3 months of treatment and 1 month after discontinuation of treatment, and TFBUT was significantly shorter 1 month after discontinuation compared to during treatment [61]. Dry eye symptoms and unstable tear film may therefore persist at least temporarily after discontinuation of isotretinoin despite reversal of meibomian gland changes. Two cases of dry eye symptoms lasting more than two years after isotretinoin cessation have been reported [70], which has been reported to occur in 1% of patients [71]. Generally, isotretinoin does not seem to affect aqueous production, as the majority of studies reported no significant difference in Schirmer’s test before, during and after isotretinoin therapy [56,58,59,61,72,73,74,75]. Although Polat et al. reported a significant difference in Schirmer’s test after starting isotretinoin, this was likely confounded by a lack of standardisation in the follow-up duration [76]. Therefore, patients on isotretinoin with DED likely experience evaporative DED rather than aqueous-deficient DED.

### 4.4. Corneal Abnormalities

Corneal structural changes associated with isotretinoin therapy include alterations in corneal thickness, corneal steepening, and stromal remodelling [77,78,79]. Ozyol et al. demonstrated corneal remodelling characterised by stromal thickening and epithelial thinning during isotretinoin treatment [78], while Yuksel et al. reported reductions in central corneal thickness [79]. These changes appear reversible following termination of treatment [80]. These corneal effects are of particular clinical relevance in patients considering refractive procedures such as LASIK, as isotretinoin-associated ocular surface dysfunction and corneal remodelling may adversely affect surgical planning, corneal healing, and postoperative visual outcomes [77]. The impact on biometry for cataract surgery of isotretinoin is not known.

### 4.5. Neuro-Ophthalmic Side Effects

Other reported side effects include neuro-ophthalmic manifestations. Cases of idiopathic intracranial hypertension (IIH) associated with isotretinoin use have been reported [81,82]. Fraunfelder et al. reported 179 of such cases associated with isotretinoin use, with a mean time to diagnosis of 2.3 months after drug exposure [83]. However, incidence data among isotretinoin users and the general population is sparse, and pharmacoepidemiologic studies are recommended. Patients typically present with headache, nausea and bilateral optic disc swelling (papilledema). Untreated permanent visual field loss can follow. A hypothesised mechanism involves isotretinoin structural and metabolic similarity to vitamin A, a known cause of IIH. In addition, concomitant use with tetracycline-class antibiotics, another established risk factor for IIH, may potentiate this risk [83], and concurrent administration is generally discouraged. The lack of robust of re-challenge data precludes a definitive causal relationship [84]. Cases of retrobulbar optic neuritis presenting with acute vision loss have also been reported in association with isotretinoin, which was found to be reversible following drug cessation [85]. This could potentially be due to an alteration in action potential propagation and synaptic transmission by isotretinoin rather than demyelination [86].

## 5. Discussion

This review highlights the considerable variation in the strength of evidence supporting different ophthalmic adverse effects of isotretinoin (Table 5). While some complications are consistently demonstrated across multiple clinical studies, others remain supported primarily by isolated reports and small observational studies. Appreciating these differences is important for clinicians when counselling patients and determining the need for ophthalmic referral.

The current literature is strongest for ocular surface complications, where findings have been reproduced across multiple independent cohorts using both objective clinical measurements and patient-reported outcomes [51,52,59,61,63,74,87]. By contrast, evidence supporting retinal structural changes remains inconsistent [41,42], while most neuro-ophthalmic complications are derived from case reports and pharmacovigilance data [81,82]. Consequently, the level of certainty differs substantially between the various ocular manifestations, and these differences should be recognised when interpreting the available evidence. Several methodological limitations likely account for the heterogeneity observed across studies. Most investigations involve relatively small sample sizes, short follow-up periods, and heterogeneous outcome measures, limiting meaningful comparison between studies [75]. Differences in cumulative isotretinoin dose, treatment duration, baseline ocular surface status, imaging techniques, and the timing of ophthalmic assessment further complicate interpretation. In addition, many studies did not adequately account for confounding factors such as pre-existing acne- or rosacea-associated MGD, contact lens wear, or concurrent medications, all of which may influence ocular findings independently of isotretinoin exposure [62,63].

An important observation emerging from this review is the disconnect between structural, functional, and symptomatic outcomes. Objective anatomical changes do not consistently correlate with symptom severity, while patient-reported symptoms may persist despite apparent structural recovery following treatment cessation [52]. This highlights the multifactorial nature of isotretinoin-associated ocular toxicity and suggests that reliance on any single investigation may underestimate disease burden. From a clinical perspective, the available evidence supports a multidisciplinary approach rather than routine discontinuation of isotretinoin in all patients who develop ocular symptoms. Most ocular complications can be recognised early and managed conservatively with appropriate ophthalmic input, allowing continuation of treatment where clinically appropriate. Conversely, the development of acute visual loss, new nyctalopia, or symptoms suggestive of raised intracranial pressure should prompt urgent ophthalmic assessment because of the potential for permanent visual sequelae despite their rarity. Important knowledge gaps remain. Prospective longitudinal studies with standardised ocular assessments before, during, and after isotretinoin therapy are needed to better define the incidence, reversibility, and long-term outcomes of ocular adverse effects. Future research should also seek to identify patient-level risk factors, establish dose–response relationships where they exist, and determine whether targeted ophthalmic screening strategies improve clinical outcomes.

Overall, the available evidence supports a clear association between isotretinoin and ocular surface toxicity, while evidence for less common posterior segment and neuro-ophthalmic complications remains comparatively limited. As isotretinoin continues to be widely prescribed, collaboration between dermatologists, general practitioners, and eye care practitioners will remain central to early recognition, appropriate management, and preservation of visual function.

## 6. Practice Points for the Dermatologist and General Practitioner

**Figure 2:**
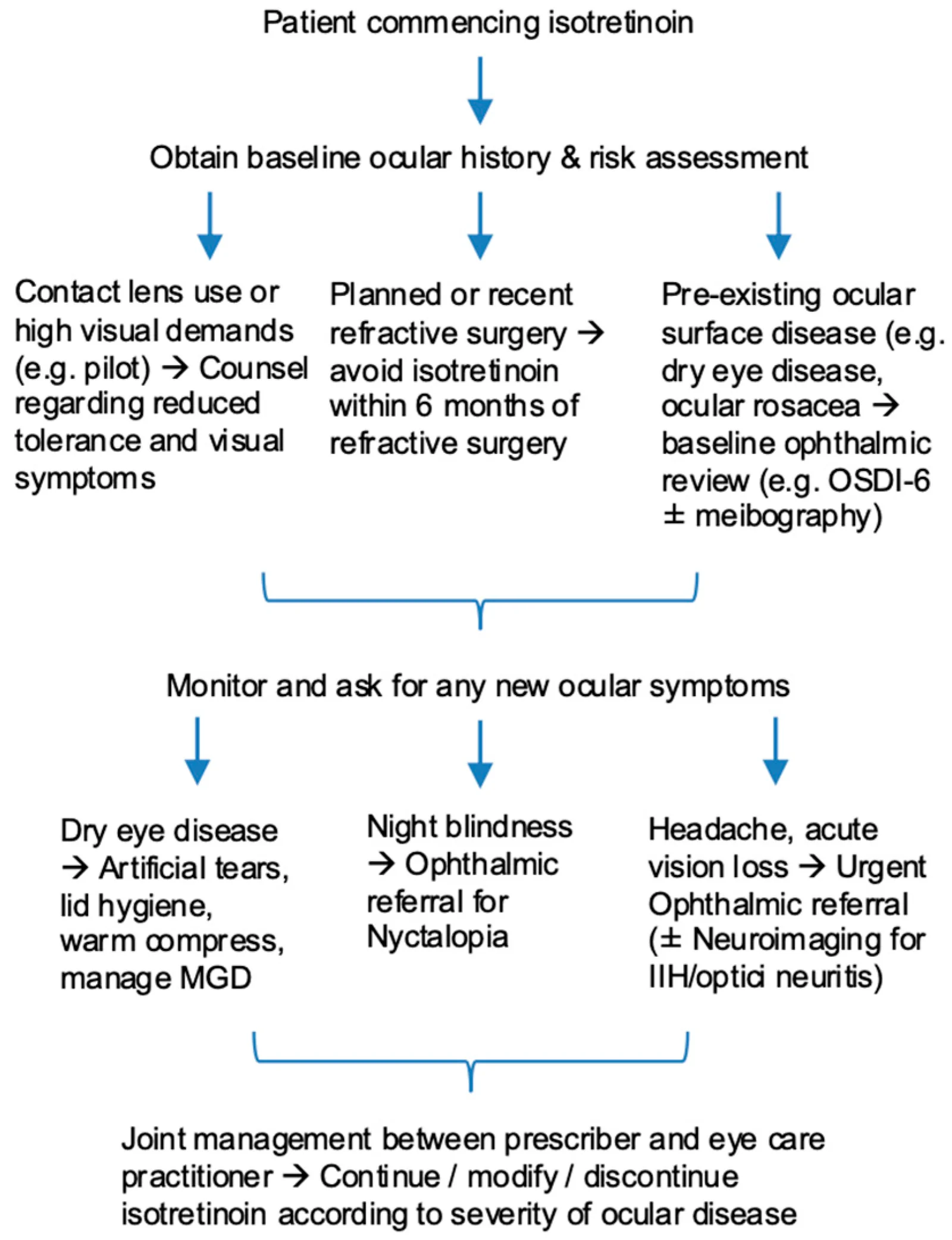
Ophthalmic evaluation of patients newly started on isotretinoin.

Isotretinoin has clear and well documented effects on night vision, meibomian gland characteristics and the ocular surface, as well as some rare potentially vision threatening complications. Prescribers should therefore be vigilant and routinely ask patients on isotretinoin if they are experiencing visual symptoms (Figure 2). The temporal relationship and associated symptoms are important to elucidate in order to differentiate night blindness from blurring of vision due to DED, as blurring of vision is commonly reported in patients on isotretinoin [37]. While patients with nyctalopia report poor dark adaptation, patients with DED also often report ocular surface discomfort, foreign body-like sensation, tearing and worsening of symptoms in gusty and cold environments [67]. Unlike DED, there is evidence to suggest the severity of nyctalopia is not only dose-dependent, but also potentially reversible [33]. Thus, any complaint of night blindness should be taken seriously and warrants urgent ophthalmic referral (Table 6). Furthermore, the severity of nyctalopia worsens in the setting of malabsorptive states or hypovitaminosis A [34], and dermatologists and GPs are in a better position to serially monitor serum vitamin A status in such patients.

Since dry eye symptoms do not necessarily correlate with the severity of MGD [59], a prompt referral to an eye care practitioner should be made if DED is suspected to allow for a more comprehensive evaluation of meibomian gland characteristics (e.g., meibography). Nevertheless, the OSDI remains a useful tool in monitoring disease progression [56]. While no treatments directly reverse meibomian gland damage, supportive management options exist [52]. The mainstay of symptomatic relief in DED is artificial tears [88]. In accordance with TFOS DEWS III [68] recommendations, a range of tear substitute formulations are available (e.g., sodium hyaluronate [89,90]), with selection guided by the underlying tear film deficiency and patient factors. In severe MGD, higher viscosity gels and ointments can be prescribed by an ophthalmologist [88,91]. More recently, lipid- and perfluorohexyloctane (PFHO)- containing drops have shown potential in the management of MGD by improving tear film stability [92,93]. Additionally, all patients on isotretinoin with MGD should be taught lid hygiene techniques to improve meibum secretion [94]. When performed adequately, maintaining good lid hygiene can potentially reverse meibomian gland dropout [95]. Since patients with acne vulgaris (the main indication for isotretinoin) and rosacea are predisposed to MGD [62,63,69], an assessment of dry eye symptoms and an early ophthalmic evaluation can be done before the initiation of isotretinoin for the identification of pre-existing MGD. In patients with concomitant rosacea, management should also include identification and avoidance of individual triggers, together with conservative ocular surface measures such as regular blinking during prolonged visual tasks, environmental modification, lid hygiene, and the use of wraparound sunglasses to reduce tear evaporation and wind exposure [96]. Pre-existing dry eye symptoms are known to worsen with isotretinoin [97]. While isotretinoin should be avoided or prescribed with caution in such patients, there is no evidence to suggest that the severity of DED symptoms is dose-dependent [97]. Prescribers should therefore continue to adhere to dosing guidelines for the relevant clinical indication should isotretinoin be initiated [4]. Since recent studies suggest reversibility of MGD with discontinuation of treatment [61], dermatologists may be inclined to stop isotretinoin if patients begin to develop DED. However, this decision should ideally be a joint decision between the eye care practitioner and prescriber based on clinical severity of DED rather than the duration of treatment, as OSDI scores have not been shown to be duration-dependent either [97]. Clinicians prescribing isotretinoin should routinely enquire about ocular and visual symptoms, with targeted ophthalmic assessment and co-management for patients who develop significant or persistent symptoms or have relevant ocular risk factors [61,70,71].

A comprehensive past ocular history and general medical history should be sought in patients starting isotretinoin as this will significantly affect the management. For instance, contact lens users should be counselled that they may experience decreased tolerance of contact lenses after starting isotretinoin [29]. A history of previous refractive procedures should also be sought in all patients before starting isotretinoin [77], and isotretinoin should be avoided 6 months before or after refractive surgery to prevent cornea ulceration or infection [98]. While most dermatologists are well aware of DED and MGD as a side effect and are even comfortable prescribing artificial tears as and when necessary, many do not elicit and are unaware of importance of a history of previous refractive surgery [99]. These aspects of the past ocular history are important as many patients who use isotretinoin are 18 to 30 years old (i.e., population prone to moderate-to-severe acne vulgaris [100]), which overlaps with the population keen for contact lenses and refractive surgery [77].

## 7. Future Perspectives

Despite increasing recognition of isotretinoin-associated ocular adverse effects, important gaps remain in defining their incidence, dose–response relationships, reversibility, and long-term outcomes. Future studies should prioritise prospective longitudinal cohorts incorporating standardised ocular assessments before, during, and after isotretinoin therapy to distinguish treatment-related changes from pre-existing ocular surface disease. Particular attention should be given to identifying patient-level risk factors, including pre-existing MGD, acne or rosacea severity, contact lens use, and nutritional or malabsorptive states. Standardisation of imaging modalities and functional outcome measures would also improve comparability between studies.

Further mechanistic research is needed to clarify how isotretinoin affects meibomian gland biology, retinal photoreceptor function, and corneal remodelling, and whether biomarkers can identify patients at increased risk of ocular toxicity. Finally, prospective studies evaluating the effectiveness of baseline ophthalmic screening and symptom-based monitoring strategies could help determine which patients would benefit most from routine eye care assessment. These data would facilitate more individualised risk stratification and multidisciplinary management while avoiding unnecessary discontinuation of an otherwise effective therapy.

## 8. Conclusions

Isotretinoin remains an invaluable tool in the dermatologist’s and GP’s arsenal. Co-management with an eye care provider is recommended, given the well documented ocular side effects, to guide initiation and discontinuation of treatment. Clinicians should maintain a high index of suspicion when patients receiving isotretinoin develop new visual symptoms, including night blindness, blurred vision, visual field disturbances, diplopia, or persistent headache with visual symptoms. While nyctalopia is a well-established adverse effect, especially in the setting of malabsorptive states, any new visual complaint should prompt formal ophthalmic evaluation. MGD can be adequately managed by the prescriber in mild cases, and the OSDI can be used to monitor dry eye symptom burden over time. Ophthalmic evaluation should be sought in patients with pre-existing DED, a history of refractive surgery or contact lens users wishing to commence isotretinoin treatment, as well as patients experiencing intolerable side effects wishing to stop treatment and patients with persistent dry eye symptoms after discontinuation of isotretinoin.

## Figures and Tables

**Table 1:** Non-exhaustive side effect profile of isotretinoin.

Body System	Common Documented Adverse Effects
Mucocutaneous [3,8,9,10]	Inflammation of the lips (i.e., cheilitis), dry skin (i.e., xerosis), transient worsening of acne vulgaris, photosensitivity, pruritus, desquamation, acne fulminans
Obstetric [11,12,13]	Congenital malformations, spontaneous miscarriage
Metabolic [14,15,16,17]	Hyperlipidaemia, metabolic syndrome, transaminitis
Musculoskeletal [19,20,21]	Hyperostosis, premature epiphyseal closure
Gastrointestinal [22,23]	Inflammatory bowel disease
Psychiatric [24,25,26]	Major depressive disorder, suicide
Ocular [27,28,29,30]	Dry eye disease, blepharoconjunctivitis, nyctalopia

**Table 2:** Ocular manifestations of Isotretinoin. Key: RNFL = retinal nerve fibre layer.

Anatomical Location	Manifestations
Meibomian Glands	Gland dysfunction
	Decreased gland density
	Gland atrophy
Cornea	Stromal thickening
	Epithelial thinning
	Opacities
Retina	Nyctalopia
	RNFL thinning
	Rod–cone dysfunction
Neuro-ophthalmic	Idiopathic intracranial hypertension
	Retrobulbar optic neuritis

**Table 3:** Effects of isotretinoin on retinal layers.

Retinal Structure	Reported Isotretinoin Associated Findings	Evidence
Retinal nerve fibre layer (RNFL)	Temporal and lower temporal RNFL thinning reported on OCT	Inconsistent findings across studies; several studies demonstrated no significant change
Ganglion cell-inner plexiform layer (GCIPL)	GCIPL thinning reported following systemic isotretinoin therapy	Limited and inconsistent evidence
Photoreceptors	Rod–cone dysfunction with abnormal ERG responses and impaired dark adaptation	Supported by electrophysiological studies
Retinal vasculature	Central retinal vein occlusion, pre-macular haemorrhage	Case reports

Key: RNFL = Retinal nerve fibre layer, GCIPL = Ganglion cell-inner plexiform layer.

**Table 4:** Evaluation of meibomian gland structure and function.

Assessment Tool	Parameters
Meibomian Gland Loss (MGL)	Quantifies the degree of meibomian gland atrophy or dropout observed on meiboraphy (graded on a scale of 0 to 3 by the area of gland loss as a fraction of total meibomian gland area)
Lid Margin Abnormality Score (LAS)	Evaluates structural lid margin abnormalities including telangiectasia, irregularity, gland orifice plugging, and displacement of the mucocutaneous junction (graded on a scale of 0 to 4, with a score of 2 or higher considered abnormal)
Meibum Expressibility Score (MES)	Assesses the ease with which meibum can be expressed from the glands (graded on a scale of 0 to 3)
Meibum Quality Score (MQS)	Grades the quality and consistency of expressed meibum, ranging from clear fluid to thickened secretions (graded on a scale of 0 to 3)

**Table 5:** Strength of evidence supporting different ophthalmic adverse effects of isotretinoin.

Ocular Manifestation	Overall Evidence Synthesis	Evidence Base	Areas of Agreement	Controversies/Knowledge Gaps	Clinical Implications
Meibomian gland dysfunction (MGD)	Strong evidence that isotretinoin induces structural and functional meibomian gland changes, leading to evaporative DED [51,52,59,61,63,74].	Consistent (multiple prospective observational studies)	MGD, reduced meibum quality, gland loss, tear film instability occur during treatment.	Degree and timeline of reversibility; relationship between gland morphology and symptoms.	Baseline assessment in high-risk patients; lid hygiene and lubricants; ophthalmic referral if symptomatic.
Tear film stability	Well-established adverse effect predominantly secondary to MGD rather than aqueous tear deficiency [56,59,62].	Consistent	Increased OSDI scores, reduced TBUT, minimal effect on Schirmer’s testing.	Persistence of symptoms after cessation; optimal monitoring strategy.	Routine enquiry regarding dry eye symptoms; manage conservatively with tear substitutes and ocular surface therapy.
Nyctalopia	Well-established functional retinal adverse effect attributable to rod photoreceptor dysfunction [33,34,35].	Consistent	ERG abnormalities and impaired dark adaptation consistently reported; generally reversible.	True incidence, dose–response threshold, predictors of irreversible dysfunction.	Prompt ophthalmic referral; important counselling for occupations requiring night vision (e.g., pilots).
Retinal structural changes	Evidence for RNFL and GCIPL thinning remains inconsistent [41,42].	Conflicting	No consensus regarding OCT changes.	Clinical significance, reproducibility, mechanism, long-term outcomes.	Routine OCT screening not currently supported.
Corneal abnormalities	Corneal remodelling appears to occur during treatment and may affect refractive surgery planning [77,78,79].	Limited-moderate	Alterations in corneal structure appear largely reversible after cessation.	Magnitude of effect, impact on refractive outcomes, implications for cataract biometry.	Delay elective refractive surgery; assess ocular surface pre-operatively.
Idiopathic intracranial hypertension (IIH)	Rare but potentially vision-threatening complication [81,82].	Limited (case reports/pharmacovigilance)	Association recognised clinically; concomitant tetracyclines may increase risk.	Incidence, causality, patient risk factors.	Urgent referral for headache, papilloedema or visual symptoms.
Optic neuritis	Very rare adverse event reported in isolated cases [85].	Very limited	Visual recovery usually follows cessation of isotretinoin.	Mechanism and causal relationship remain uncertain.	Immediate ophthalmic assessment for acute vision loss.

**Table 6:** Practical recommendations for clinicians prescribing isotretinoin.

Clinical Issue	Recommended Action	Evidence Basis
Dry eye/MGD	Consider ophthalmic referral for persistent or significant symptoms; assess meibomian glands and tear-film function. Supportive measures include artificial tears and lid hygiene.	Guideline directed (TFOS DEWS III)
New nyctalopia	Urgent ophthalmic assessment. Assess for contributing factors including vitamin A deficiency/malabsorption.	Supported by consistent evidence and clinical safety consideration
Acute visual loss/severe headache	Urgent ophthalmic/neuro-ophthalmic assessment to exclude optic neuritis or raised intracranial pressure.	Clinical safety consideration
Refractive surgery	Establish history of previous/planned corneal or refractive surgery and coordinate management with the ophthalmologist.	Clinical interpretation
Continuing isotretinoin	Ocular symptoms do not automatically require discontinuation. Decisions should be individualised according to severity and made jointly with the prescriber and eye care practitioner.	Clinical interpretation

## Data Availability

No new data were created or analyzed in this study. Data sharing is not applicable to this article.
